# Heredity

**Published:** 1886-03-20

**Authors:** 


**Heredity.**—The late Professor Laycock (American Practioner and News) was very fond of drawing attention to hereditary peculiarities. One time, in the middle of a lengthened exposition of the features in common of a mother and child, the woman, perhaps a little uneasy, stopped him, saying, " A weel ! I'm no the bairn's mither ; I'm just his stepmither."

